# *malT *knockout mutation invokes a stringent type gene-expression profile in *Actinobacillus pleuropneumoniae *in bronchoalveolar fluid

**DOI:** 10.1186/1471-2180-9-195

**Published:** 2009-09-14

**Authors:** Abdul G Lone, Vincent Deslandes, John HE Nash, Mario Jacques, Janet I MacInnes

**Affiliations:** 1 Department of Pathobiology, Ontario Veterinary College, University of Guelph, Guelph, Ontario N1G 2W1, Canada; 2 Groupe de Recherche sur les Maladies Infectieuses du Porc, Université de Montréal, St-Hyacinthe, Québec J2S 7C6, Canada; 3 Centre de Recherche en Infectiologie Porcine, Université de Montréal, St-Hyacinthe, Québec J2S 7C6, Canada; 4 Institute for Biological Sciences, National Research Council of Canada, Ottawa, Ontario K1A 0R6, Canada; 5 Office of Biotechnology, Genomics and Population Health, Public Health Agency of Canada, Ottawa, Ontario K1A 0K9, Canada

## Abstract

**Background:**

*Actinobacillus pleuropneumoniae *causes contagious pleuropneumonia, an economically important disease of commercially reared pigs throughout the world. To cause this disease, *A. pleuropneumoniae *must rapidly overcome porcine pulmonary innate immune defenses. Since bronchoalveolar fluid (BALF) contains many of the innate immune and other components found in the lungs, we examined the gene expression of a virulent serovar 1 strain of *A. pleuropneumoniae *after exposure to concentrated BALF for 30 min.

**Results:**

In reverse transcription PCR differential display (RT-PCR DD) experiments, *A. pleuropneumoniae *CM5 exposed to BALF up-regulated, among other genes, a gene predicted to encode LamB, an outer-membrane transport protein of the maltose regulon. To determine the role of the *lamB *and other genes of the maltose regulon in the pathogenesis of *A. pleuropneumoniae*, knockout mutations were created in the *lamB *and *malT *genes, the latter being the positive transcriptional regulator of the maltose regulon. Relative to the *lamB *mutant and the wild type, the *malT *mutant had a significant (*P *< 0.05) decrease in growth rate and an increased sensitivity to fresh porcine serum and high concentrations (more than 0.5 M) of sodium chloride. In DNA microarray experiments, the BALF-exposed *malT *mutant exhibited a gene-expression profile resembling that of a stringent type gene-expression profile seen in bacteria facing amino acid or carbon starvation. Genes encoding proteins for protein synthesis, energy metabolism, and DNA replication were down-regulated, while genes involved in stringent response (e.g., *relA*), amino acid and nucleotide biosynthesis, biofilm formation, DNA transformation, and stress response were up-regulated.

**Conclusion:**

These results suggest that MalT may be involved in protection against some stressors and in the transport of one or more essential nutrients in BALF. Moreover, if MalT is directly or indirectly linked to the stringent response, an important global mechanism of bacterial persistence and virulence in many bacterial pathogens, it might play a role in *A. pleuropneumoniae *pathogenesis.

## Background

*A. pleuropneumoniae *causes contagious pleuropneumonia in pigs. The disease can occur in acute, sub-acute, or chronic form [1]. The acute form is characterized by fibrinohemorrhagic pneumonia and the sub-acute and chronic forms by pleuritis with localized necrotizing lesions. The severity and the spread of the disease depend upon the serovar and dose of the strain, and in large measure, upon the immune status of the herd [2].

*A. pleuropneumoniae *is well adapted to survive and replicate in the host respiratory tract. Its survival and replication requires the expression of genes encoding proteins that protect the bacterium from the host immune response and help it to acquire nutrients. Although RTX (repeats in toxin) toxins, lipopolysaccharide, capsule, and various amino acid and iron transport systems of the bacterium are essential to cause acute disease [3], it is not known how the organism survives in the face of non-cellular innate immune components that form the first line of defence in the lungs [4]. To identify *A. pleuropneumoniae *genes that are expressed in a medium that mimics, at least in part, the alveolar surface environment of the lungs, we incubated the bacterium in concentrated porcine bronchoalveolar lavage fluid (BALF). In addition to innate immune components, such as collectins, defensins, lysozyme, lactoferrin, and cathelicidin [4], BALF contains surfactant, surfactant-associated proteins, dissolved minerals, and other substances functioning in antioxidation, lipid metabolism, and tissue repair and proliferation in the lungs [5]. Thus, genes expressed by *A. pleuropneumoniae *in porcine BALF may be important for survival and pathogenesis of the organism.

In RT-PCR DD experiments, *A. pleuropneumoniae *CM5 exposed to BALF for 30 min differentially expressed a number of genes, including seemingly a *lamB *homolog. Consistent with this finding, an earlier study had also reported that *A. pleuropneumoniae *expresses a maltose-inducible, LamB-like outer membrane protein in the host[6]. In *E. coli *and other gram-negative bacteria, *lamB *encodes an outer membrane transport protein involved in the transport and metabolism of maltose and maltodextrins. The *E. coli *maltose regulon is comprised of at least ten genes whose transcription is positively regulated by MalT in the presence of maltotriose derived from either imported maltodextrins or endogenous glycogen [7].

In addition to maltose and maltodextrin transport and metabolism, the genes of the maltose regulon have been associated, in ways less well understood, with virulence in bacteria. For example, MalF, an inner membrane maltose and maltodextrin transport protein, and MalQ, a dextrinyl transferase, have been associated with the expression of cholera toxin and toxin-co-regulated pilus in *Vibrio cholerae *[8], as has been LamB with cytopathic effect in enteropathogenic *E. coli *[9], and adhesion in enteroinvasive *E. coli *[10] and *Aeromonas veronii *[11]. Mutants of the *malE *and *malT *(transporter) genes in group A *Streptococcus *are attenuated in their ability to grow in human saliva and to metabolize α glucans and are significantly impaired in their ability to colonize the mouse oropharynx [12,13].

To elucidate the role of the predicted maltose regulon in *A. pleuropneumoniae*, *malT *and *lamB *knockout mutants were constructed and characterized phenotypically. Since MalT is a regulatory protein, the effect of its knockout on the bacterial gene expression level was also determined using DNA microarrays.

## Results

### Expression of maltose-regulon genes by the wild-type *A. pleuropneumoniae *CM5 in BALF

**Figure 1:**
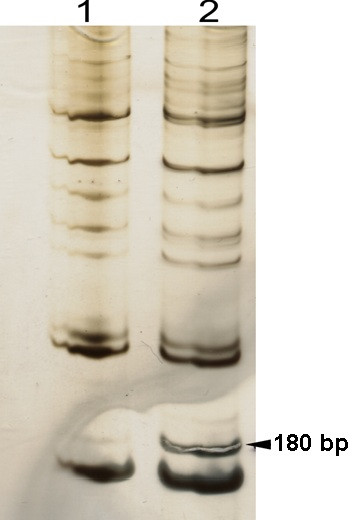
**Silver-stained gel comparing *A. pleuropneumoniae *RT-PCR DD products in BHI broth (1) and BALF (2)**. The arrow points to the band representing a differentially expressed gene, which based on cloning and sequencing (see Methods), appeared to be *lamB*.

Several differentially expressed genes in *A. pleuropneumoniae *CM5 exposed to BALF for 30 min at 37°C were first presumptively identified by RT-PCR DD studies. These included genes encoding protein synthesis and hypothetical proteins (APL_068, APL_0363, and APL_0367), in addition to a cell surface protein, LamB (Figure 1). Homologs (>99% DNA identity) of the 3 hypothetical proteins are present in all the serotypes of *A. pleuropneumoniae *sequenced so far, suggesting that they might have a role in persistence or pathogenesis, but their levels of expression were not confirmed by real-time PCR or other more direct methods. The level of expression of the *lamB *gene was estimated by real-time PCR analysis to be 3.3-fold higher in BALF- than in BHI-exposed cells (Table 1). Genes of the maltose regulon that were also up-regulated (although some at very low levels) in BALF-exposed cells included *malF *and *malG *(encoding the intrinsic membrane proteins of maltose transport system), *malP *(maltodextrin phophorylase), *malQ *(amylomaltase) and *malK *(the ATP-binding cassette of the maltodextrin transporter; Table 1). For further study, we constructed *lamB *and *malT *mutants to evaluate the possible role of these genes in the survival of *A. pleuropneumoniae *CM5.

**Table 1:** Differential expression of maltose-regulon genes in BALF-exposed *A. pleuropneumoniae *CM5

Gene	Putative function	ΔΔC_T _± SD	Fold-change*
*malE *(T)	Periplasmic maltose binding protein	-2.82 ± 0.51	7.06 (4.95-10.05)
*malE *(R)		0 ± 0.84	1 (0.55-1.79)
*malF *(T)	Intrinsic membrane protein of maltose transport system	-2.79 ± 1.01	6.91 (3.43-13.92)
*malF *(R)		0 ± 0.39	1 (0.76-1.31)
*malG *(T)	Intrinsic membrane protein of the maltose transport system	-2.6 ± 0.40	6.06 (8-4.59)
*malG *(R)		0 ± 0.40	1(0.76-1.31)
*malK *(T)	ATP-binding protein of the maltodextrin transporter	-1.10 ± 0.39	2.14 (1.6-2.8)
*malK *(R)		0 ± 0.76	1(0.59-1.69)
*lamB *(T)	Maltoporin	-1.73 ± 0.46	3.31 (2.41-4.56)
*lamB *(R)		0 ± 0.35	1(0.78-1.27)
*malP *(T)	Maltodextrin phosphorylase	-0.85 ± 0.46	1.80(1.31-2.46)
*malP *(R)		0 ± 0.79	1(0.58-1.72)
*malQ *(T)	Amylomaltase	-0.96 ± 0.48	1.94(1.39-2.71)
*malQ *(R)		0 ± 0.55	1(0.68-1.46)
*malT *(T)	Transcriptional activator of maltose-regulon genes	-0.75 ± 0.32	1.68(1.34-2.09)
*malT *(R)		0 ± 0.79	1(0.58-1.72)

* Fold change is the fold increase or decrease in the level of expression of a gene in the wild type exposed to BALF (target sample, abbreviated as T) relative to the level of expression of the gene in the wild type exposed to BHI (calibrator or reference sample, abbreviated as R), as measured by real-time PCR.

Values in the parentheses represent the range in the fold change.

### Growth curves of the *malT *and *lamB *mutants

**Figure 2:**
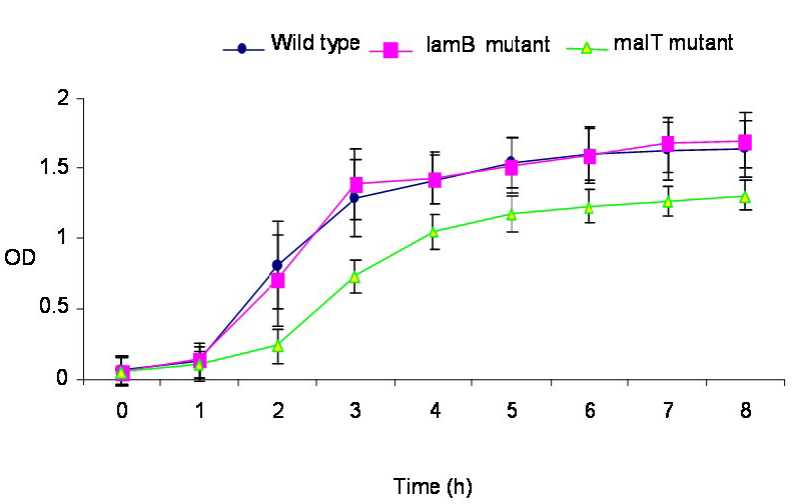
**Growth curves of the wild type strain and *lamB *and *malT *mutants in BHI broth**.

The *malT *mutant grew slower than the wild-type organism in BHI. The growth pattern of the *lamB *mutant was, however, similar to that of the wild-type organism (Figure 2).

### Effect of acarbose on the growth of the isogenic *malT *and *lamB *mutants of *A. pleuropneumoniae *CM5

**Figure 3:**
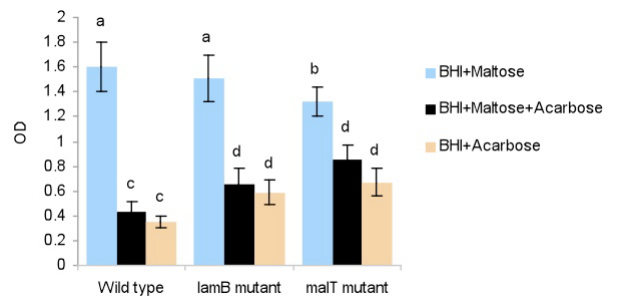
**Overnight growth of the wild type strain and the *lamB *and *malT *mutants in acarbose or maltose**. The bars with same letters on the top do not differ significantly (*P *< 0.05)

To assess the effect of the *malT *knockout mutationon the functioning of the maltose regulon, the parent strain and the *malT *mutant were grown in acarbose-containing BHI in the presence or absence of maltose. Acarbose is a competitive inhibitor of maltose transport [14]. Because of the fastidious nutritional requirements of *A. pleuropneumoniae*, this experiment was performed in BHI instead of a chemically defined medium. After 16 h of incubation in acarbose-containing BHI that was supplemented with maltose, the wild-type organism reached a significantly lower OD_600 _(*P *< 0.05) than did the *malT *mutant (Figure 3). In acarbose-containing BHI that was not supplemented with maltose, there was again, a significant difference in the growth of the two strains. The number of wild type and *malT *mutant cells was lower in acarbose-containing BHI than in the BHI containing both maltose and acarbose; however, this difference was not significant (Figure 3). The *lamB *mutant showed a trend similar to that of the *malT *mutant grown in the acarbose-containing medium, but the number of *lamB *mutant cells was lower than that of the *malT *mutant; however, this difference was not significant.

### Survival of the *malT *and *lamB *mutants

**Figure 4:**
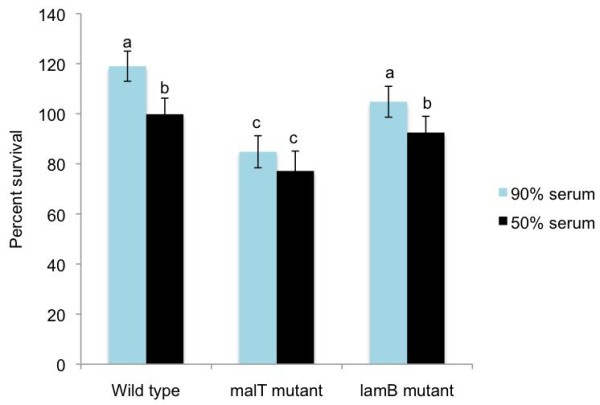
**Percent survival of the wild type strain, and the *malT *and *lamB *mutants in porcine serum**. The percent survival is the fresh-serum-surviving CFU expressed as the percent of CFU surviving in the heat inactivated serum. The strains were incubated in fresh and heat-inactivated serum for 1 h. The bars with same letters on the top do not differ significantly (*P *< 0.05)

Because LamB is a cell surface protein that is positively regulated by MalT, we examined the effects of serum and high concentrations of sodium chloride to better understand the role of these genes in the survival of *A. pleuropneumoniae*. The percent survival of the *malT *mutant after incubation at 37°C for 1 h in 90 and 50% porcine serum was significantly (*P *< 0.05) lower than the percent survival of the wild- type strain (Figure 4). There was no significant difference in the survival between the wild-type organism and the *lamB *mutant in either concentration of the serum. The number of cells of all the three strains (wild-type organism, *malT *and *lamB *mutants) surviving in 90% serum was higher than the number of cells surviving in 50% serum. *E. coli *DH5α did not survive in either concentration of serum.

**Figure 5:**
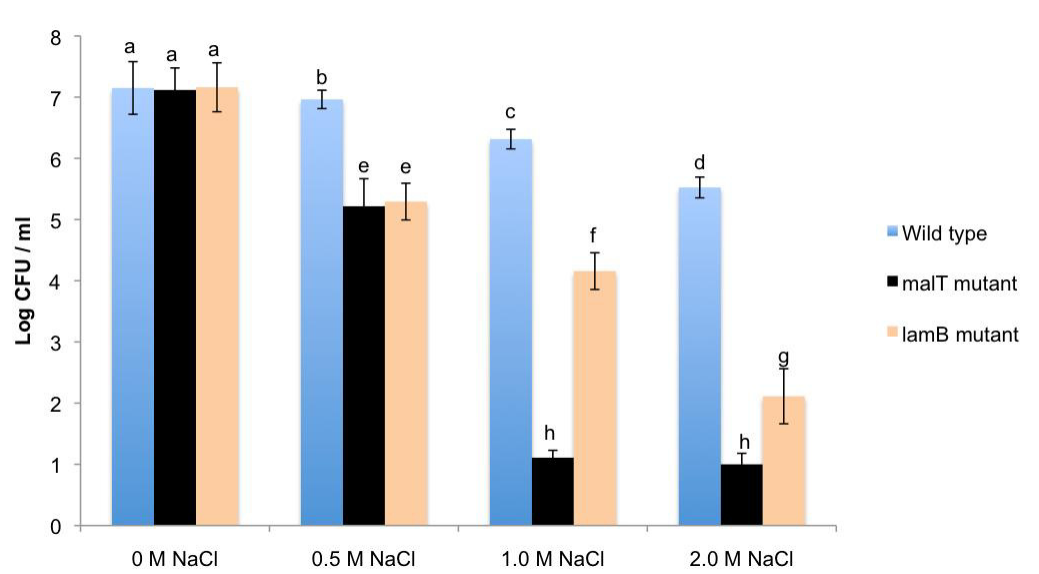
**CFU of the wild type strain, and the *malT *and *lamB *mutants in different NaCl concentrations**. The strains were incubated for 3 h in the salt-containing BHI medium. Before being exposed to NaCl, the strains were grown in maltose-containing BHI. The bars with the same letters on the top do not differ significantly (*P *< 0.05)

In the maltose-supplemented BHI containing different concentrations of sodium chloride, the wild type parent, and the *malT *and *lamB *mutants showed a significant (*P *< 0.05) decrease in cell numbers after 3 h of incubation (Figure 5). The decrease in the cell number was least in the wild-type organism and greatest in the *malT *mutant. In 1 M sodium chloride, the *malT *mutant decreased in number from an initial count (prior to the addition of the salt to the medium) of 10^7 ^CFU/ml to a final count (3 h subsequent to the addition of the salt to the medium) of 10 CFU/ml. Even at a 2 M salt concentration, the wild-type organism decreased in number to only 5 log CFU/ml from approximately the same initial count as that of the *malT *mutant. At salt concentrations of 1 M and above, the *lamB *mutant showed a decline in cell numbers midway between those of the numbers shown by the parent strain and the *malT *mutant. The wild-type organism, and the *malT *and *lamB *mutants were all susceptible to killing by high concentrations of sodium chloride, but this killing was greatest in the *malT *mutant (Figure 5).

### Differential gene expression by the *malT *mutant in BALF

To understand the basis of the observed phenotypic differences between the *malT *mutant and the wild-type organism, gene expression profiles of the mutant and parent strains were compared using DNA microarrays. Following the incubation of the exponentially grown cultures of the mutant and wild-type organism in fresh BHI at 37°C for 30 min, no significant differences were observed in the gene expression profiles of the two strains even at low delta values. Incubation in BALF, however, resulted in a total of 223 genes being differentially expressed in the *malT *mutant at a false discovery rate (the percentage of the differentially expressed genes identified just by chance) of 1%. The differentially expressed genes included 104 up-regulated and 119 down-regulated genes and 92 of these encoded hypothetical proteins (Table 2, Additional file 1: Analyzed microarray data). In general, the genes encoding proteins involved in energy metabolism and protein biosynthesis were down-regulated (Table 3), while as those involved in amino acid and nucleotide biosynthesis, DNA transformation, and biofilm formation were up-regulated (Table 4). The *relA *gene encoding a stringent response regulatory protein was also up-regulated in the *malT *mutant. Though known as an *in vivo*-expressed RTX toxin, the *apxIVA *gene was up-regulated by the wild-type strain in BALF [15] and its expression was further increased in BALF in the *malT *mutant.

**Table 2:** Number of genes expressed differentially* within a functional category by the BALF-exposed *malT *mutant

Functional category	Up-regulated genes	Down-regulated genes
Protein biosynthesis	2	7
Amino acid biosynthesis	6	2
Cofactor biosynthesis	4	8
Biofilm formation	4	0
Nucleotide biosynthesis	3	0
Lipid biosynthesis	0	2
Lipid degradation	1	0
Cell envelope biosynthesis	3	10
Cellular processes	5	2
Central intermediary metabolism	0	4
DNA metabolism	3	6
Energy metabolism	7	18
Protein folding and stabilization	2	2
Regulatory proteins	7	2
Transcriptional regulators	0	5
Secretion and trafficking	4	10
Mobile and extra-chromosomal function	2	0
Unclassified and unknowns	51	41
Total	104	119

* Differential expression of a gene in the *malT *mutant is relative to the level of expression of the gene in the wild-type organism, as measured in microarray experiments.

**Table 3:** Protein-synthesis and energy-metabolism genes expressed differentially* by the BALF-exposed *malT *mutant

Type of the product encoded by the differentially expressed gene	Up-regulated genes	Down-regulated genes
Ribosomal proteins and their modifiers	*rpmE*	*rplQ*, *rpsQ*, *rplI*, *rpmG*
tRNA base modifiers	*queA*	*alaS*, *truD*, *trmU*,
Transcription and transcription-related factors		*deaD*, *rnc*, *rph*, *nusA*, *nusB*
Amino acid biosynthetic enzymes	*trpD*, *dapA*, *argD*, *proC*, *leuC*, *ilvH*, *tyrR*	*aroA*, *aroB*,
Periplasmic nitrate reductase (*nap *operon)		*napB*, *napG*, *napF*, *napD*, *napH*
Nitrite reductase (nrf operon)		*nrfB*, *nrfC*
Dimethyle sulfoxide reducatse (*dms *operon)		*dmsA*, *dmsB*
Hydrogenases		*hyaA*, *hybB*
Amino acid catabolism		*sdaA*, *aspA*
pyruvate formate-lyase 1-activating enzyme		*PflA*
Glycolysis and gluconeogenesis	*gpmB*, *pepC*	*FruK*
TCA cycle enzymes	*sucD*, *lpdA*	
Non-glucose hexose-monosaccharide metabolism enzymes	*mtlD*	*nagZ*
Products of central intermediary metabolism		*ureA*, *ppx*
ATP synthase		*atpC*, *atpB*
Formate dehydrogenase		*fdhE*
Products involved in fermentation	*dld*, *aldA*	
Regulatory proteins	*narP*, *sixA*, *gntR*, *cysB*, *asnC*, *gcvA*, *rseA*	*arcA*, *iclR*
Cofactors	*folA*, *folP*, *pdxY*, *thiH*	*hemB*, *chuW*, *lipA*, *ispA*, *ddc*, *dxs*, *ispE*, *iscA*

* Differential expression of a gene in the *malT *mutant is relative to the level of expression of the gene in the wild-type organism (reference sample) as measured in microarray experiments. For complete gene names and the fold changes in gene expression see Additional file 1: Analyzed microarray data.

**Table 4:** Nutrient-acquisition, replication and virulence genes expressed differentially* by the BALF-exposed *malT *mutant

Type of the product encoded by the differentially expressed gene	Up-regulated genes	Down-regulated genes
Biofilm-formation proteins	*pgaA*, *pgaC*, *tadF*, *apfB*	
Toxin	*apxIVA*	
Factors imparting resistance to antimicrobials		*ostA*, *ccp*
Peptidoglycan and LPS biosynthetic enzymes	*cpxD*, *mrdA*	*dacA*, *murA*, *mltA*, *dacB*, *mreD*, *fbB1*, *kdsB*, *gmhA*
Membrane proteins	*ompP1*	*ompW*, *oapB*
Amino acid transporters		*brnQ*, *sdaC*
Carbohydrate transporter	*mtlA*	*ptsB*, *rbsD*
Iron transport proteins	*cbiO*	*exbD2*, *afuB_2*, *frpB*, *yfeC*, *exbB2*
Protein/peptide transport proteins	*dppF*	
Other cation transporters		*ptsN*
Cell division	*fic*	
Lipid transporters	*glpF*	
Factors involved in adaptation to unusual environment	*relA*	
DNA transformation	*comEA*, *comF*	
DNA degradation proteins	*xseA*	
DNA replication, recombination proteins	*recG*, *rdgC*, *recJ*	*xerC*, *recR*, *priB*, *polA*, *ligA*, *recA*,
Protein-fate proteins	*htpX*, *prlC*	*ecfE*
Nucleotide metabolism enzymes	*purC*, *purD*, *purT*	
Phopholipid and fatty acid biosynthesis and degradation enzymes	*namA*	*accA*, *fabD*

* Differential expression of a gene in the *malT *mutant is relative to the level of expression of the gene in the wild-type organism (reference sample). For complete gene names and the fold changes in gene expression see Additional file 1: Analyzed microarray data.

Expression of selected genes representing biological functional categories of interest was also measured by real-time PCR analysis (Table 5). A good corroboration in the context of the up- and down-regulation of the genes was found between the microarray and real-time PCR data.

**Table 5:** Verification of microarray data by real-time PCR

Gene	Putative function	ΔΔC_T _± SD	Fold change by real-time PCR	**Fold change by microarray**^1^
*dmsA *(T)	Anaerobic dimethyl sulfoxide reductase chain A precursor	3.45 ± 1.41	0.091 (0.03-0.24)	0.15
*dmsA *(R)		0 ± 0.51	1 (0.69-1.42)	
*dmsB *(T)	Anaerobic dimethyl sulfoxide reductase chain B	2.54 ± 1.61	0.17 (0.05-0.52)	0.34
*dmsB *(R)		0 ± 0.46	1 (0.72-1.38)	
*napB *(T)	Nitrate reductase cytochrome c-type subunit	2.24 ± 0.41	0.21 (0.15-0.28)	0.17
*napB *(R)		0 ± 0.49	1 (0.71-1.40)	
*napF *(T)	Ferredoxin-type protein NapF	2.24 ± 0.46	0.21 (0.07-0.61)	0.09
*napF *(R)		0 ± 0.47	1 (0.71-1.39)	
*napD *(T)	Putative napD protein	2.39 ± 0.34	0.18 (0.14-0.24)	0.18
*napD *(R)		0 ± 0.54	1 (0.68-1.46)	
*ilvH *(T)	Acetolactate synthase small subunit	-2.60 ± 0.36	6.08 (4.68-7.90)	6.14
*ilvH *(R)		0 ± 0.45	1 (0.70-1.41)	
*pgaA *(T)	Biofilm PGA synthesis protein PgaA precursor	-2.04 ± 1.08	4.11 (1.94-8.70)	8.18
*pgaA *(R)		0 ± 0.74	1 (0.59-1.67)	
*pgaC *(T)	Biofilm PGA synthesis N-glycosyltransferase PgaC	-2.47 ± 0.42	5.54 (4.12-7.45)	6.23
*pgaC *(R)		0 ± 1.05	1(0.48-1.07)	
*apxIVA *(T)	RTX toxin protein	-3.01 ± 1.12	8.06 (3.69-17.61)	6.5
*apxIVA *(R)		0 ± 0.60	1 (0.65-1.52)	
*relA *(T)	GTP pyrophosphokinase	-0.95 ± 0.42	2.0 (1.44-2.56)	6.30
*relA *(R)		0 ± 0.59	1(0.66-1.51)	
*lamB *(T)^2^	Maltoporin	1.03 ± 0.39	0.49 (0.37-0.64)	na^3^
*lamB *(R)		0 ± 0.23	1 (0.85-1.17)	

^1^Fold change is the fold increase or decrease in the level of expression of a gene in the *malT *mutant (target sample, abbreviated as T) relative to the level of expression of the gene in the wild type (calibrator or reference sample, abbreviated as R) in BALF except for the *lamB *gene^2 ^whose expression was compared in BHI to examine the effect of the *malT *knockout mutation on the expression of the *lamB *gene. ^3 ^Not applicable.

Values in the parentheses represent the range in the fold change.

## Discussion

### Expression of maltose-regulon genes by BALF-exposed *A. pleuropneumoniae *CM5

After exposure of *A. pleuropneumoniae *CM5 to BALF for 30 minutes, a gene that appeared to be *lamB *homologue was shown to be up-regulated by the organism in RT-PCR DD experiments (Figure 1). We selected 30 min for incubation of the organism in BALF, as the medium conditions should remain fairly constant during this time as might be seen in the animal during early infection when there is constant replenishment of alveolar fluid. As shown in real-time PCR studies, the genes encoding intrinsic membrane transport system proteins (MalF and MalG), maltodextrin phosphorylase (MalP), amylomaltase (MalQ), ATP-binding cassette of the maltodextrin transporter (MalK) of the maltose regulon were also up-regulated in BALF, although some at very low levels (Table 1). Comparison of gene expression in BALF- and BHI-incubated cells by DNA microarrays [15] showed that *malF *and *malG *were up-regulated in BALF. However, no differential expression was seen in *malT*, *malK*, *malP *or *malQ *genes. This disparate finding could be because only small quantities of these proteins are required for function, and small changes in gene expression are difficult to detect. For further study, we focused on the *lamB *and *malT *genes of the maltose regulon as LamB is a cell surface protein that lies at the host-pathogen interface and MalT is a transcriptional regulator that might control the expression of genes other than those involved in the maltose and maltodextrin transport and metabolism.

### *malT *and *lamB *are the components of a functional maltose regulon in *A. pleuropneumoniae *CM5

All of the strains of *A. pleuropneumoniae *sequenced so far possess homologs of the maltose regulon genes *malEFG*, *malK-lamB-malM*, *malT *and *malPQ*. As demonstrated by microarray-based comparative genomic profiling, these genes are present in the reference strains of all 15 serovars of *A. pleuropneumoniae *[16]. It might be noted that maltose regulon genes are also present in two other upper respiratory tract pathogens, *Mannheimia haemolytica *and *Haemophilus parasuis*. The arrangement of some of these genes in *A. pleuropneumoniae*, however, differs from that found in *E. coli*. As in *E. coli*, MalT appears to be a positive transcriptional regulator of *lamB *in *A. pleuropneumoniae *as demonstrated by a two-fold decrease in the expression of *lamB *in the isogenic *malT *mutant of *A. pleuropneumoniae *CM5 in BHI supplemented with maltose (Table 5). This finding is consistent with an earlier phenotypic study [6] which reported that *A. pleuropneumoniae *expresses a LamB-like outer membrane protein when maltose is added to BHI agar. Moreover, the *A. pleuropneumoniae *MalT and LamB has a high degree of amino acid similarity with MalT and LamB homologs of a number of other Gram-negative organisms. Also, MalT has a conserved DNA-binding (LuxR-like C-terminal containing helix-turn-helix) motif such as found in the *E. coli *MalT protein.

To further examine the effect of the *malT *mutation on the regulation of the maltose regulon, both the wild-type organism and the *malT *mutant were grown in the presence of acarbose. Acarbose is a pseudo-oligosaccharide similar in structure to maltotetraose and it is a competitive inhibitor of maltose transport in *E. coli*. It can inhibit maltose uptake only if maltose-transport system is first activated by maltose. Acarbose also inhibits α-amylases and α-glucosidases and is not degraded by *E. coli *[14]. In BHI supplemented with maltose, acarbose reduced the growth of the wild-type organism as well as that of the *malT *mutant (Figure 3). The reduction in the growth might have been caused either by accumulation of toxic levels of acarbose by the bacterial cells or by the inhibition of bacterial glucosidases by the accumulating acarbose, or both. The reduction was, however, significantly (*P *< 0.05) greater in the wild-type organism than in the mutant. This is perhaps due to the increased uptake of acarbose by the wild-type organism, owing to its higher activation of the maltose regulon by the intact *malT*. On the other hand, the reduction in the growth of the *malT *mutant could have been due to the non-specific entry of acarbose into the bacterial cells.

As *A. pleuropneumoniae *CM5 is not amenable to complementation it should be noted that we can not rigorously exclude the possibility that the phenotype exhibited by the *malT *negative strain was affected by some alteration of another gene that occurred during strain construction, but this is very unlikely. That said, taken together, the above findings suggest that *A. pleuropneumoniae *has a functional maltose regulon similar to that of *E. coli*.

### *malT *is required for optimum survival of *A. pleuropneumoniae *CM5 in serum and high concentrations of sodium chloride

In comparison with the wild-type *A. pleuropneumoniae *CM5 and *lamB *mutant, the *malT *mutant had a significantly decreased ability to survive following incubation in fresh porcine serum for 1 h; the wild-type organism, however, grew in serum to a significantly higher number (Figure 4). As resistance of *A. pleuropneumoniae *to killing by serum is predominantly due to its capsule and LPS [17,18], the decreased survival of the *malT *mutant in serum could have been due to a change in its cell surface polysaccharides or to an alteration in its general metabolism as indicated by its slower growth in BHI. Similarly, in the presence of sodium chloride concentrations of more than 0.5 M, the *malT *mutant had a significantly (*P *< 0.05) diminished ability to survive in the BHI supplemented with maltose. This result suggests that MalT-regulated genes are required for protection against the high concentrations of sodium chloride in *A. pleuropneumoniae *(Figure 5). An association has been shown to exist between the components of the maltose regulon, stress response, and hypersomolarity in *E. coli *[19], but it is not known how the maltose regulon behaves in the presence of an exogenous activator and high concentrations of the sodium chloride.

### Differential gene expression of the *malT *mutant in BALF resembles the stringent type gene-expression profile

There was no significant difference between the gene expression profile of the parent strain and the *malT *mutant after incubation of the log-phase cultures in fresh BHI for 30 min. In BALF, however, 223 genes were differentially expressed by the *malT *mutant (Table 2). The gene expression profile of the mutant resembled a metabolic downshift; genes encoding protein synthesis, energy metabolism, transport of nutrients and DNA replication were all down-regulated, while those involved in amino acid and nucleotide biosynthesis, biofilm formation (prevalent in *A. pleuropneumoniae *field isolates [20]), DNA transformation, and the stress response were up-regulated (Tables 3 and 4). This type of gene-expression response mimics the gene-expression profile of the stringent response seen in *E. coli *and other organisms during nutrient deprivation [21-23].

Carbon starvation in *E. coli *invokes a global gene expression response, resulting in the down-regulation of the genes encoding proteins for the growth and replication of the organism and the up-regulation of the genes encoding proteins for the biosynthesis of amino acids, alternate sigma factors, biofilm components [24], as well as proteins of unknown function [25]. During amino acid starvation, the ratio of uncharged to charged tRNA increases, resulting in ribosome stalling at the A-site of the 50S ribosomal subunit. The stalling of the ribosome results in the activation of ribosome-bound RelA. RelA, a synthase and SpoT, a hydrolase with a weak synthase activity, synthesize pppGpp (guanosine 3'-diphosphate,5'-triphosphate) and ppGpp (guanosine 3', 5'-bispyrophosphate) which in turn invoke a global gene expression response including down-regulation of rRNA synthesis, such as seen in the stringent response to nutrient starvation [24].

The increased expression of *relA *and the changes in the overall gene expression profile of the *malT *mutant in BALF closely resembled the stringent-response gene- expression profile in other bacteria, including *E. coli*. Consistent with the notion of a stringent response having a role in *A. pleuropneumoniae*, all the major stringent response regulatory genes including *relA*, *spoT *and *dksA *(DnaK suppressor protein) are present in the genome of this pathogen. A *malT *knockout mutation in *A. pleuropneumoniae *could result in a stringent response because MalTis linked, directly or indirectly, to the regulation of the stringent response genes, or because it regulates the uptake of nutrient(s) in addition to maltose and maltodextrins. The latter assumption could explain the up-regulation of the *lamB *gene in BALF as a secondary response to the activation or the up-regulation of MalT for the acquisition of nutrients. The slower growth of the *malT *mutant and its increased sensitivity to the biological stressors could also be explained by changes in cell surface molecules that result from the inability of the mutant to acquire unknown essential nutrient(s). By balancing nutrient availability with ribosome synthesis through the stringent response, bacteria can control replication, enter into a persistence mode of life, or express virulence factors, depending upon the type of bacteria [26-29].

## Conclusion

Taken together, our data suggest that *A. pleuropneumoniae *CM5 has a functional maltose regulon similar to that found in *E. coli*. Although it is likely that these genes have a role in acquisition of nutrients in saliva and in the oropharynx where maltodextrins would be predicted to be found, these studies suggest that the maltose regulon could also play a significant role once the organism enters the lungs. Further, the slower growth rate and increased salt and serum sensitivity of the *malT *mutant versus *lamB *mutant suggests that MalT has a role beyond that of maltose and maltodextrin metabolism in *A. pleuropneumoniae*. This is perhaps due to the involvement of the MalT in the transport or processing of some essential nutrient(s). This assumption is further supported by the expression of the stringent type transcript profile in the *malT *mutant in BALF. MalT could also be directly or indirectly linked to the stringent response without being involved in the transport of the essential nutrient(s); however, this remains to be proven. The presence of the maltose-regulon genes in all serovars of *A. pleuropneumoniae *and in related pathogens such as *Mannheimia haemolytica *and *Haemophilus parasuis *provides further circumstantial evidence that carbohydrate metabolism mediated by the maltose regulon might play a role in the persistence, if not the pathogenesis of some respiratory tract pathogens.

## Methods

### Bacterial strains and media

*A. pleuropneumoniae *CM5 [30], and *E. coli *strains β2155 [31] and DH5α (Clontech, CA, USA) were used in this study (Tables 6 and 7). *A. pleuropneumoniae *CM5 was grown either in brain heart infusion (BHI; Becton, Dickinson and Company, Sparks, MD, USA) or Mueller-Hinton (MH; Becton, Dickinson and Company) medium, supplemented with 0.01% (wt/vol) β-nicotinamide adenine dinucleotide (NAD) as required. Transconjugation medium consisted of MH broth with 20% (wt/vol) sucrose, 10% equine serum (wt/vol), and 0.01% NAD (wt/vol). *E. coli *strains were routinely cultured in Luria-Bertani (LB) medium, but in the case of *E. coli *β2155, the medium was always supplemented with 1 mM diaminopimelic acid (DAP; Sigma-Aldrich, St. Louis, MO, USA). As required, chloramphenicol was also added at the rate of either 5.0 or 2.5 μg/ml.

**Table 6:** Bacterial strains, plasmids and primers used in the construction of the *malT *mutant

Bacterial strains, plasmids or primers	Characteristic or sequence	Source or Remark
*E. coli *DH5a	F-φ80*lac*ZΔM15Δ(*lac*ZYA-*rg*F)U169 *deo*R *rec*A1 *end*A1 *hsd*R17(rk -, mk +) *sup*E44 *thi*-1 *gyr*A96 *rel*A1 λ-	Clonetech
*E. coli *β2155	*thr*B1004*pro thi hsdS lacZΔ*M15 (F'lacZΔM15*lacI*^q ^*traD*36 *proA*^+ ^*proB*^+^) Δ*dap*::*erm*(Erm^r^)*recA*::RP4-2-*tet*(Tc^r^)Mu- km(Km^r^)λpir	Reference no. 28
*E. coli *DH5α-pTOPOPCR-malT	DH5α harboring pCR4-TOPO containing *malT *of *A. pleuropneumiae *CM5	This work
*E. coli *DH5α- pTopoMC	DH5a harboring pCR4-TOPO containing Δ*malT*::*cat*	This work
*E. coli *DH5α-pEMOC2M	DH5a harboring pEMOC2 containing Δ*malT*::*cat*	This work
*A. pleuropneumoniae*	MalT negative mutant of *A*.	This work
CM5 3ΔmalT	*pleuropneumonaie *CM5	
pCR4-TOPO	A linearized plasmid for cloning PCR product	Invitrogen
pEMOC2	A conjugation vector based on pBluesript SK with *mob*RP4 and Cm^r^	Reference no. 31
pTOPOPCR-malT	pCR4-TOPO containing *malT *of *A. pleuropneumiae *CM5	This work
pTopoMC	pCR4-TOPO containing Δ*malT*::*cat*	This work
pEMOC2M	harboring pEMOC2 containing Δ*malT*::*cat*	This work
**malT-L** malT-R	ATGCAAGCAACATTTTCAAGA TTAGCTATACCCCATCATTCTCAA	Primers for amplification of the *malT *gene of *A* *pleuropneumoniae CM5*
**stopupmalT-L**	**TTAGTTAGTTACGAGCTTTTTCACACCGTTT**	Primers for generation of a linearized plasmid containing a deletion of 900 bp in its *malT *gene cloned in pTOPOPCR-malT.
stopupmalT-R	TAACTAACTAATGGGAATGGCATCATTTAGA	
**pnmalT-L**	**TCATCTGCAGATGCAAGCAACATTTTCAAGA**	Primers for amplication of the Δ*malT*::*cat *and the insertion of the *Pst*I *and Not*I sites into the PCR product.
pnmalT-R	ACAATACAGCGGCCGCTTAGCTATACCCCATCATTCTCAA	
**cat-L**	**CGGTGCCCTGAATGAACT**	Primers for the PCR
cat-R	AAGCTTCGACGAATTTCTGC	amplification of *omlA-P *driven *cat *gene of pEMOC2

**Table 7:** Bacterial strains, plasmids and primers used in the construction of the *lamB *mutant

Bacterial strains, plasmids or primers*	Characteristic or sequence	Source or Remark
*E. coli *DH5α-pTOPOFL	DH5α harboring pCR4-TOPO containing *lamB *of *A. pleuropneumia e *CM5	This work
*E. coli *DH5α-TOPOΔFLcat	DH5a harboring pCR4-TOPO containing Δ*lamB*::*cat*	This work
*E. coli *DH5Δ-pEMOC2-ΔlamB	DH5Δharboring pEMOC2 containing Δ*lamB*::*cat*	This work
*A. pleuropneumoniae *CM5 ΔlamB	LamB negative mutant of *A. pleuropneumoniae *CM5	This work
pTOPOFL	pCR4-TOPO containing *lamB *of *A. pleuropneumiae *CM5	This work
TOPOΔFLcat	pCR4-TOPO containing Δ*lamB*::*cat*	This work
pEMOC2-ΔlamB	pEMOC2 containing Δ*lamB*::*cat*	This work
**CrosslamB-L** CrosslamB-R	**GGTGGCGTAAAAGTAGGAGAT** TGGTCATTATCCACCACCAA	Primers for the PCR amplification of the *lamB *gene of *A. pleuropneumoniae CM5*
**stopuplamB-L**	**TTAGTTAGTTACAATATTTTCAACCCCTGCAC**	Primers for the PCR generation of a linearized plasmid containing a deletion of 400 bp in the *lamB *gene cloned in pTOPOPCR-lamB
stopuplamB-R	TAACTAACTAATCACGCACAAGGTTC AAAAG	
**PstcrosslamB-L** NotcrosslamB-R	**TCATCTGCAGGGTGGCGTAAAAGTAGGAGAT** ACAATACAGCGGCCGCTGGTCATTATCCACCACCAA	Primer sequences for the PCR amplication of the Δ*lamB*::*cat *and the insertion of the *PsT*I *and Not*I sites into the PCR product

* The genotype and the source of *E. coli *DH5α and the pEMOC2 and pCR4-TOPO plasmids are given in Table 6.

### Collection and concentration of bronchoalveolar lavage fluid

BALF was collected from ten high-health status pigs of approximately 15 kg in body weight. After euthanizing the pigs, the lungs of the individual animals were lavaged with 100 ml of PBS (phosphate-buffered saline), and the lung washings were collected and centrifuged to remove cell debris. The contents of the washings were then concentrated with a 5 kDa molecular weight cut off ultra-centrifugal filter device, Vivacell 70 (Vivascience Ltd., Stonehouse, GL, UK), which reduced the volume of the washings to 1/20^th ^that of their total initial volume. The concentrated BALF was sterilized by filtration through a 0.22 μm membrane filter (Pall Corporation, Ann Arbor, MI, USA) and kept at -80°C for long-term storage. Molecules less than 5 kDa in molecular weight were not concentrated by this method; nevertheless, the fluid still contained these substances in the concentrations found before ultrafiltration.

### Reverse-transcription PCR differential display

The RT-PCR DD method described by McClelland et al. [32] was adapted to identify the differentially expressed genes of *A. pleuropneumoniae *CM5 in BALF. Briefly, the organism was grown to an OD_600 _of 0.7 in BHI at 37°C, harvested by centrifugation, and an approximately 10^7 ^colony forming units (CFU) were suspended in either concentrated BALF or fresh BHI. After incubation of the cell suspensions at 37°C for 30 min, the bacteria were harvested by centrifugation and immediately subjected to RNA extraction.

RNA was extracted with Trizol reagent (Invitrogen, Carlsbad, CA, USA) and quantified using RNA 6000 Nano LabChip chips read in a Bioanalyzer 2100 instrument (Agilent Technologies, Santa Clara, CA, USA). The RNA was treated with Turbo RNA-free DNase (Ambion Inc., Austin, TX, USA) according to the manufacturer's instructions.

A total of 0.5 μg of RNA and 85 different combinations (Table 8) of arbitrary random primers (GenHunter Corp., Nashville, Tennessee, USA) (Table 9) were used to synthesize cDNA with Moloney Murine Leukemia Virus reverse transcriptase (M-MLV reverse transcriptase; Invitrogen). Reverse transcriptase-negative controls were run with each of the transcription reaction.

**Table 8:** Arbitrary random primer pair combinations used in RT-PCR DD

AP17/AP18	AP17/AP19	AP17/AP20	AP17/AP21	AP17/AP21
AP17/AP21	AP17/AP22	AP17/AP23	AP17/AP24	AP17/AP24
AP17/AP24	AP18/AP18	AP18/AP19	AP18/AP19	AP18/AP20
AP18/AP20	AP18/AP21	AP18/AP21	AP18/AP22	AP18/AP22
AP18/AP23	AP18/AP23	AP18/AP24	AP19/AP18	AP19/AP20
AP19/AP21	AP19/AP22	AP19/AP23	AP19/AP23	AP19/AP24
AP20/AP18	AP20/AP21	AP20/AP22	AP20/AP23	AP20/AP24
AP21/AP24	AP21/AP18	AP21/AP22	AP21/AP23	AP22/AP18
AP22/AP23	AP22/AP24	AP23/AP18	AP23/AP24	AP24/AP18
AP41/AP18	AP41/AP42	AP41/AP43	AP41/AP44	AP41/AP45
AP41/AP46	AP41/AP47	AP41/AP48	AP42/AP18	AP42/AP43
AP42/AP44	AP42/AP45	AP42/AP46	AP42/AP46	AP42/AP47
AP43/AP18	AP43/AP44	AP43/AP45	AP43/AP46	AP43/AP47
AP43/AP48	AP43/AP48	AP44/AP18	AP44/AP45	AP44/AP46
AP44/AP47	AP44/AP48	AP45/AP18	AP45/AP46	AP45/AP46
AP45/AP47	AP45/AP48	AP46/AP18	AP46/AP47	AP46/AP48
AP47/AP18	AP47/AP48	AP47/AP48	AP47/AP48	AP48/AP18

**Table 9:** Sequences of the arbitrary random primers used in RT-PCR DD

Arbitrary random primer	Sequence
AP17	AAGCTTACCAGGT
AP18	AAGCTTAGAGGCA
AP19	AAGCTTATCGCTC
AP20	AAGCTTGTTGTGC
AP21	AAGCTTTCTCTGG
AP22	AAGCTTTTGATCC
AP23	AAGCTTGGCTATG
AP24	AAGCTTCACTAGC
AP41	AAGCTTACGGGGT
AP42	AAGCTTTGCACCG
AP43	AAGCTTGAAGCGG
AP44	AAGCTTCTCCGGA
AP45	AAGCTTGGCTGAC
AP46	AAGCTTCGGTCCT
AP47	AAGCTTATGCCCG
AP48	AAGCTTGCGGTGA

One microlitre of the reverse-transcription reaction mixture was used as a template to amplify the cDNA under relaxed PCR conditions. The same primer pairs were used in both the template cDNA synthesis and the random PCR -amplification of the template cDNA. The 20-μl PCR reaction mixtures contained 1.5 μM of each of the forward and reverse primers, 2.0 μl of 10 × PCR buffer, 200 μM of dNTP mixture, 4.0 mM MgCl2, and 2.5 U of *Taq *DNA polymerase (New England Biolabs, Pickering, ON, Canada). The PCR thermal profile included an initial random primer annealing and extension steps (denaturation 94°C for 5 min; primer annealing at 39°C for 5 min; and strand extension at 72°C for 3 min) followed by a 40-cycle PCR (denaturation 95°C for 2 min; primer annealing at 39°C for 2 min; and strand extension at 72°C for 1 min) with a final amplification step of 10 min at 72°C. PCR products of the same primer pair were run side by side on 7% polyacrylamide gels and silver stained, as described elsewhere [33], to visualize the bands representing differentially expressed genes (Figure 1). Bands representing differentially expressed genes were scratched with a 25 gauge needle to harvest DNA. The DNA on the pointed end of the needle was dissolved in a 10 μl of PCR-grade water for 5 min. This solution of DNA served as a template for a PCR reaction in which the same protocol and the same primers were used as in the differential display PCR that generated the band. The amplified DNA was run on agarose gels and stained with ethidium bromide to visualize the bands for excision. The DNA from the excised bands was purified using QIAquick Gel Extraction Kits (Qiagen Inc., Mississauga, ON, Canada), and the purified PCR products were cloned into the pCR4-TOPO (TOPO TA Cloning Kit, Invitrogen), according to the manufacturer's instructions. The inserts were sequenced by dye terminator cycle sequencing (DNA Sequencing Facility, College of Biological Sciences, University of Guelph, Guelph, ON) and compared with the annotated genome sequences of *A. pleuropneumoniae *using Blastx available at http://blast.ncbi.nlm.nih.gov/Blast.cgi to identify the complete genes.

### Construction of the *malT *knockout mutant

Based on the genome sequence of *A. pleuropneumoniae *serovar 1 strain 4074, primers were designed to amplify the entire *malT *gene (nucleotides 2118860 to 2121577). The *malT *PCR product was purified and cloned into pCR4-TOPO. The resultant plasmid was used as the template in a PCR reaction to produce a linearized plasmid with a deletion of the central 838 bp (bp 922 to bp 1760) of the *malT *gene. The amplicon was generated using Phusion *Taq *DNA polymerase (New England Biolabs), a high fidelity DNA polymerase, and the primers that annealed in back to back manner leaving a central 900 bp region of the plasmid *malT *between them. Following the gel purification of the PCR product, the *omlA-P *promoter driven chloramphenicol acetyl transferase gene (*cat*), obtained by PCR amplification of pEMOC2 [34] was blunt-end ligated with the linear plasmid. The resultant circular plasmid with the *cat *insertion in the *malT *was designated as pTopoMC. The Δ*malT*::*cat *fragment of pTopoMC was then PCR amplified with forward and reverse primers containing *Not*I and *Pst*I sites, respectively. The Δ*malT*::*cat *PCR amplicon was gel purified, digested with *Not*I and *Pst*I, and cloned into pEMOC2. The resultant plasmid, named pEMOC2M, was electroporated into *E. coli *β2155. pEMOC2M was mobilized from *E. coli *β2155 into *A. pleuropneumoniae *CM5 using a modification of the filter mating technique described by Oswald et al. [35]. Briefly, overnight cultures of *E. coli *β2155/pEMOC2M (grown on LB agar containing 25 μg/ml chloramphenicol), and *A. pleuropneumoniae *CM5 (grown on BHI agar) were washed with 2 ml of TNM buffer (1 mM Tris-HCl, pH 7.2; 10 mM MgSO4; 100 mM NaCl). The OD_600 _of both the donor and the recipient strains was adjusted to 1 by adding TNM buffer. A 100 μl volume of the donor and 10 μl of the recipient strains were mixed by inversion, and the mixture was centrifuged to pellet the cells, which were washed and then resuspended in 1 ml of fresh TNM buffer. A 50 μl volume of the suspension was spotted onto a 0.45 μm nitrocellulose filter (Pall Corporation) placed onto the BHI agar plate containing DAP and MgSO_4 _(10 mM). After incubation at 37°C for 6 h in an atmosphere of 5% CO_2_, the filter was washed with 5 ml of BHI broth. The cells were harvested by centrifugation and re-suspended in 0.5 ml of BHI broth. After 10-fold serial dilution of the cell suspension, 50 μl of cells from each of the dilution was plated onto BHI agar plates containing chloramphenicol (5 μg/ml). After 24 h of incubation at 37°C, the individual colonies appearing on the agar plates were inoculated in 1 ml of MH broth for further incubation at 37°C for 3 h. The cell suspensions of each of the colony were plated on the MH plates containing 2.5 μg/ml chloramphenicol. These plates were incubated at 29°C for 48 h. A few colonies from each of the plates were used in colony PCR to verify the integration of the plasmid into the chromosomal *malT *geneof *A. pleuropneumoniae *CM5. The primers for the colony PCR were designed so that one primer annealed inside the integrated plasmid and the other on the nearby bacterial chromosomal DNA, thus verifying both plasmid integration and orientation.

The colonies that had undergone plasmid integration at the correct site were selected for the sucrose counter-selection. Selected individual colonies with an integrated plasmid were incubated with constant agitation in 1 ml of MH broth at 37°C until the cultures were slightly turbid. A 1 ml volume of the counter-selection medium was then added and each of the cultures was incubated for a further 5 h. A 50-μl cell suspension from each of the ten-fold serial dilutions (10^0 ^to 10^7^) of these cultures was then plated onto the MH agar plates containing sucrose (10%) and chloramphenicol (2.5 μg/ml). After incubation at 37°C for 48 h, colonies appearing on the plates were patched onto two BHI agar plates; one containing chloramphenicol (2.5 μg/ml) and the other, ampicillin (100 μg/ml). Chloramphenicol-resistant, ampicillin-sensitive colonies were screened for the second crossover by the PCR using the primers that annealed to the regions of the bacterial chromosome immediately flanking the *malT *gene. The predicted disruption of the *malT *gene was confirmed by Southern blotting using the wild type *malT *gene as a probe and by sequencing the PCR amplicon spanning the *cat *gene insertion. The primers and plasmids used in the construction of the *malT *mutant are given in Table 6.

### Construction of the *lamB *knockout mutant

The construction of the *lamB *knockout mutant involved the same approach as described for the construction of the *malT *mutant. A central 379-bp region (bp 518 to bp 897) of the *lamB *was replaced with the *omlA-P *driven *cat *gene and the knockout mutation was confirmed by sequencing and Southern blotting. The primers and plasmids used in the construction of *lamB *mutant are given in Table 7.

### Growth of the mutants

*A. pleuropneumoniae *CM5, and its isogenic *malT *and *lamB *mutants were grown in BHI at 37°C to monitor their growth. The OD_600 _of each of the strains was measured every hour from the lag to stationary phase of growth to construct growth curves. For doubling time calculations, culture aliquots were taken at 2, 3, and 4 h of incubation and the number of CFUs was determined by the plating of 10-fold dilutions. The data were analyzed using one way analysis of variance (ANOVA) and the means were compared using Tukey's method.

The wild-type organism and the *malT *and *lamB *mutants were also incubated in the BHI containing 0.5% (wt/vol) acarbose and 0.5% (wt/vol) maltose to assess the effect of acarbose on the growth of these strains. As the strains grew slowly in the acarbose-containing BHI, their growth was measured after 16 h of incubation at 37°C.

### Survival of the mutants in serum

Individual colonies from the overnight cultures of *A. pleuropneumoniae *CM5, the *malT *and *lamB *mutants, and *E. coli *DH5α, were incubated in 5 ml of BHI at 37°C for 2 h. A 1 ml volume of each of the cultures was centrifuged at 10,000 ×*g *for 2 min to pellet the cells before suspension in 1 ml of pre-warmed PBS. One hundred μl of a 1:10^5 ^dilution of each culture was added to 900 μl of 100 and 55.5% fresh porcine serum (vol/vol in PBS). As a control, 100 μl of 1:10^5 ^dilution of each culture was also added to 900 μl of heat-inactivated porcine serum (inactivated by heating at 65°C for 15 min). The number of CFU of each culture was determined after the incubation of the cultures at 37°C for 1 h. The number of the CFU surviving in fresh serum was expressed as percent survival according to the following equation:

The experiment was run in quadruplicate, and the percent-survival data were divided by 2 before being converted to arcsin values for the analysis using two-way ANOVA. Means were compared by Tukey's Method.

### Survival of the mutants in sodium chloride

*A. pleuropneumoniae *CM5, and the *malT *and *lamB *mutants were grown to an OD600 of 0.7 in the BHI broth supplemented with 1% (wt/vol) maltose. Each of these cultures was mixed with fresh BHI containing 4 M sodium chloride in equal proportions for a final concentration of 2 M sodium chloride; cultures containing 1 and 0.5 M of the salt were prepared by the same approach. The number of CFU of each culture was calculated prior to the addition of the salt-containing BHI and 3 h subsequent to the incubation at 37°C in salt-containing medium. The experiment was repeated four times, and the data obtained were analyzed using ANOVA. Means were compared using Tukey's Method.

### Microarray experiments

The AppChip2 microarray chips used in this study, were an evolved version of the AppChip1 chip, and like its predecessor, was a part of the *A. pleuropneumoniae *5b L20 genome sequencing project (NRC-IBS, Ottawa, Canada). For the construction of AppChip2, open-reading-frame (ORF) PCR fragments of 160-nucleotide length and above were spotted in duplicate on the microarray slides. The spots represent 2033 ORFs, covering 95% of the total ORFS, from the complete genome sequence of the organism. Spotted sheared genomic DNA from *A. pleuropneumoniae *L20 and porcine DNA were used as controls http://ibs-isb.nrc-cnrc.gc.ca/glycobiology/appchips_e.html. Further details concerning chip production are described elsewhere [36].

Based on the strain (the wild-type organism or the *malT *mutant) and the incubation medium (BHI or BALF), the microarray experiments involved three types of hybridizations: (1) Cy3-labeled cDNA from the BHI-incubated wild-type organism vs. Cy5-labeled cDNA from the BALF-incubated wild-type organism (2) Cy3-labeled cDNA from the BHI-incubated wild-type organism vs. Cy5-labeled cDNA from the BALF-incubated *malT *mutant, and (3) Cy3-labeled cDNA from the BHI-incubated wild-type organism vs. Cy5-labeled cDNA from BHI-incubated *malT *mutant. Four replications, including dye-swaps, were carried out for each type of hybridization.

cDNA was synthesized in the presence of amino-allyl-dUTP (Sigma-Aldrich, St. Louis MO, US), random octamer primers (Biocorps, Montreal, QC, Canada), SuperScript II transcriptase (Invitrogen, Carlsbad, CA, US), and the RNA (15 μg per reaction) obtained from the BALF- and BHI-incubated organisms, according to the method described by Carrillo et al. [37]. Labeling of the cDNA was carried out indirectly with one of the mono-functional NHS-ester dyes Cy3 or Cy5 (GE Healthcare, Buckinghamshire, UK), which binds to the amino-allyl-dUTP of the cDNA. The dye labeling efficiency of cDNA was determined spectrophotometrically. The data were submitted to the Gene Expression Omnibus (GEO: GSE13006).

### Microarray data analysis

Microarray image and data analysis was carried out using the TM4 Suite of software [38] for microarray analysis, (J. Craig Venter Institute, JCVI, USA) as described elsewhere [36]. Briefly, images were analyzed with Spotfinder v3.1.1. The final intensity of each spot was obtained by subtracting the background intensity from the integral spot intensity (the sum of the intensities of all the spot pixels excluding the saturated ones). The spots with intensities less than one standard deviation above their spot background intensities were eliminated from the downstream analysis, as were the ones with total intensity less than 10000. Replicate spots were analyzed subsequent to the normalization of the data using the LOWESS (locally weighted linear regression) algorithm. The genes that were thus represented by good quality spots (defined by a score assigned by the software based on the number of unsaturated pixels, shape, and signal to noise ratio of the spot) on a minimum of two replicate slides were considered for the downstream analysis using SAM (significance analysis of microarray) to identify the differentially expressed genes. The differentially expressed genes were classified depending upon their biological roles into various functional categories according to the JCVIs Comprehensive Microbial Resources (CMR) database.

### Quantitative real-time PCR

The parameters of RNA capacity, optimum primer concentration, and the gene dynamic ranges were determined before carrying out the real-time PCR for the relative quantification of the target gene expression. As an endogenous control, the level of prolyl-tRNA-synthetase gene (*syp*) expression was used to normalize the target gene expression levels, since this gene exhibited the least variation in expression across various conditions in both the microarray and real-time PCR experiments. In the quantitative real-time PCR experiments, three independent biological replicates were tested in triplicate. Calculation of the relative quantification of the target genes was done using the Comparative C_T _(ΔΔC_T_) method [39]. The protocol of the PCR is given as described below:

Each 20-μl PCR reaction mixture contained 2 × Power SYBR Green PCR Master Mix (Applied Biosystems, Streetsville), 100 nM of each of forward and reverse primer, and 5 μl of template cDNA. Synthesis of the template cDNA was carried out in a 20-μl reaction mixture containing 500 ng RNA, using a High Capacity cDNA Reverse Transcription Kit (Applied Biosystems), which contains random primers for the synthesis of cDNA. The real-time PCR thermal profile included the heat-activation of AmpliTaq Gold DNA Polymerase at 95°C for 10 min, 40 cycles of denaturation at 95°C for 15 s, and primer annealing and extension at 60°C for 1 min. The PCR reactions were carried out in 96-well plates using a StepOnePlus thermocycler (Applied Biosystems, Streetsville, ON, Canada). The primers used in the real-time PCR are given in Table 10.

**Table 10:** Oligonucleotide primers used in the real-time PCR

Gene	Forward primer	Reverse primer
*dmsA*	ATGTTGCCGGACAAGCACAAGATG	TCTCAATGGACAACGGCTACCACA
*dmsB*	AACAGGCATCGATTGCACCGTTAC	ACTTGGACGTGCGTGTTTATTGGC
*napB*	GCGCATGGCAACCTAAACATTGGT	TACAGGCTTTGCAGTAGCGGAAAC
*napD*	TCGGCTAAAGCAAGCTGTCTGTCA	TAGCGCAAGTGAAAGCGGACATTC
*napF*	ACAACCGTCTCCGCAACTTCTACA	TTGGCTACAACGGAAGAAGCATGG
*ilvH*	GAAAGTTTAACCGTTGCGCCGACT	ACGTTCAATATGCTCGGTAGGGCT
*pgaA*	GGGAACCGGTGTGAATGCAATGAA	TGTTGGAACGTTTGTGAAGACGCC
*pgaC*	ATCGTTGCGTTACACCAAGCGAAC	ACCGACATACTTGCCTCTTGCGAT
*apxIVA*	TTGGACTTCACCTGCAAACATGCC	CGGGCAAATATTCCAAAGCGCAGA
*relA*	TCGGACAGTTGAAGTGGGAAT	TGCAAGGCGATTACTCGGTAA
*syp*	AAGAAACGCCGAATGATGCACAGG	ACACCTCGATAGCACCACCTTTGT
*lamB*	CTGCTAAAGAGAGTTTACCGATGCCA	TGCAACATTACGGGCAGGTAAACG
*malK*	GCGTGTTGCAATTGGACGTACCTT	CATGGCTTCGATTTGGTCATGCGT
*malM*	AGCGACACCGTCAAAGACAGAACT	CCAACGTTTGGCTAAATGTGCGGA
*malT*	TCCTTGATGAGCTTTCGACCCACA	TAAACCGAGCACCTGCCATTCTCT
*malP*	ACGCTTAGCCGCCTGCTATTTAGA	CACGCATCGCCTTCTTCATGTTGT
*malQ*	ATGCCTATCGGCCTTTACCGTGAT	ACCGACAGAGGCATCTAGCACAAA
*malE*	AACCGATGAAGGACTCACAACCGT	TTTCCGCATTCGCCATAGTTGCTG
*malF*	TGCCGTTAATGATTGCCAGCTTCG	GCAGCCGCTAAACCAAAGTCTTGT
*malG*	AGTGTTACTCATGCGGACGGAAGT	GCATACGCAGCAGTGGTTGAAAGT

## List of abbreviations

BALF: bronchoalveolar lavage fluid; BHI: Brain Heart Infusion; CFU: colony forming unit(s); NaCl: sodium chloride; NAD: β-nicotinamide adenine dinucleotide; ORF: Open Reading Frame; PAG: polyacrylamide gel; PCR: polymerase chain reaction; RT-PCR DD: reverse-transcription PCR differential display; vol: volume, wt: weight.

## Authors' contributions

AGL and JIM conceived and designed the experiments. AGL conducted the experiments, carried out the data analysis, and drafted the manuscript. VD carried out microarray hybridization experiments and data analysis. JHEN designed and fabricated the microarray chip, Appchip2. MJ also helped in the study design and critically revised the manuscript. All the authors contributed to the final manuscript preparation and approved its submission for publication.

## Supplementary Material

Additional file 1 **Differentially expressed genes of the BALF-exposed *A. pleuropneumoniae malT *mutant, grouped according to biological role**. Analyzed microarray data of the BALF-exposed *A. pleuropneumoniae malT *mutant. Click here for file
