# Supplementary material for: malT knockout mutation invokes a stringent type gene-expression profile in Actinobacillus pleuropneumoniae in bronchoalveolar fluid
Source: BMC Microbiol. 2009 Sep 14;9:195. doi: 10.1186/1471-2180-9-195 (PMC2752462; doi:10.1186/1471-2180-9-195)
Supplement: Additional file 1 — Differentially expressed genes of the BALF-exposed A. pleuropneumoniae malT mutant, grouped according to biological role. Analyzed microarray data of the BALF-exposed A. pleuropneumoniae malT mutant. [file 1471-2180-9-195-S1.doc]

## **Differentially expressed genes of the BALF-exposed *A. pleuropneumoniae* *malT* mutant, grouped according to biological role.**

| **ORF No**. | **Gene** | **Predicted protein** | **Fold change** |
| --- | --- | --- | --- |
| **Amino acid biosynthesis** |  |  |  |
| APL_0699 | *aroA* | 3-phosphoshikimate 1-carboxyvinyltransferase | -4.4 |
| APL_0193 | *aroB* | 3-dehydroquinate synthase | -2.3 |
| APL_0899 | *dapA* | dihydrodipicolinate synthase | 6.72 |
| APL_0244 | *argD* | acetylornithine aminotransferase (ACOAT) | 3.67 |
| APL_0160 | *proC* | pyrroline-5-carboxylate reductase | 5.82 |
| APL_0139 | *leuC* | 3-isopropylmalate dehydratase large subunit 2 | 3.46 |
| APL_0728 | *ilvH* | acetolactate synthase small subunit | 6.14 |
| APL_0797 | *tyrR* | transcriptional regulatory protein TyrR | 3.94 |
| **Biofilm formation** |  |  |  |
| APL_1921 | *pgaA* | biofilm PGA synthesis protein PgaA precursor | 8.18 |
| APL_1923 | *pgaC* | biofilm PGA synthesis N-glycosyltransferase PgaC | 6.23 |
| APL_0547 | *tadF* | tight adherence protein F | 6.29 |
| APL_0879 | *apfB* | fimbrial biogenesis protein | 4.35 |
| **Biosynthesis of cofactors** |  |  |  |
| APL_0883 | *folA* | dihydrofolate reductase | 3.64 |
| APL_1876 | *folP* | Dihydropteroate synthase | 3.87 |
| APL_1988 | *hemB* | delta-aminolevulinic acid dehydratase | -4.43 |
| APL_1523 | *chuW* | coproporphyrinogen III oxidase | -2.42 |

| **Biosynthesis of cofactors** |  |  |  |
| --- | --- | --- | --- |
| APL_1593 | *lipA* | lipoyl synthase | -7.73 |
| APL_0807 | *ispA* | geranyltranstransferase | -3.27 |
| APL_1975 | *ddc* | L-2,4-diaminobutyrate decarboxylase | -3.22 |
| APL_0207 | *dxs* | 1-deoxy-D-xylulose-5-phosphate synthase (DXPS) | -2.88 |
| APL_0776 | *ispE* | 4-diphosphocytidyl-2-C-methyl-D-erythritolkinase | -2.38 |
| APL_0929 | *iscA* | iron-binding protein IscA | -2.14 |
| APL_1485 | *pdxY* | pyridoxamine kinase | 9.78 |
| APL_0536 | *thiH* | thiazole biosynthesis protein ThiH | 2.44 |
| **Cell envelope** |  |  |  |
| APL_1596 | *dacA* | D-alanyl-D-alanine carboxypeptidase fraction A | -7.24 |
| APL_1286 | *murA* | UDP-N-acetylglucosamine1-carboxyvinyltransferase | -4.12 |
| APL_0816 | *mltA* | membrane-bound lytic murein transglycosylase A precursor | -3.23 |
| APL_0945 | *dacB* | penicillin-binding protein 4 precursor | -3.18 |
| APL_0437 | *mreD* | rod shape-determining protein MreD | -2.37 |
| APL_1559 | *mrdA* | penicillin-binding protein 2 | 4.87 |
| APL_1472 | *rfbB1* | dTDP-glucose 4,6-dehydratase | -5.13 |
| APL_0085 | *kdsB* | 3-deoxy-manno-octulosonate cytidylyltransferase | -3.12 |
| APL_1364 | *gmhA* | putative phosphoheptose isomerase | -2.69 |
| APL_1582 | *cpxD* | capsule polysaccharide export protein | 2.53 |
| APL_0933 | *ompP1* | putative outer membrane protein precursor | 4.69 |

| **Cell envelope** |  |  |  |
| --- | --- | --- | --- |
| APL_1086 | *ompW* | outer membrane protein W precursor | -15.25 |
| APL_1404 | *oapB* | opacity associated protein B | -3.41 |
| **Cellular processes** |  |  |  |
| APL_0405 | *relA* | GTP pyrophosphokinase | 6.3 |
| APL_0962 | *ostA* | organic solvent tolerance protein precursor | -2.68 |
| APL_1406 | *comEA* | DNA uptake protein | 4.15 |
| APL_2004 | *comF* | Competence protein F | 5.38 |
| APL_1379 | *ccp* | cytochrome c peroxidase | -8.65 |
| APL_0860 | *fic* | filamentation induced by cAMP protein Fic-like protein | 2.49 |
| APL_0998 | *apxIVA* | RTX toxin protein | 6.5 |
| **Central intermediary metabolism** |  |  |  |
| APL_1618 | *ureA* | urease gamma subunit UreA | -4.43 |
| APL_0708 | *ppx* | exopolyphosphatase | -3.45 |
| APL_1674 | *dmsA* | anaerobic dimethyl sulfoxide reductase chain A precursor | -6.66 |
| APL_1675 | *dmsB* | anaerobic dimethyl sulfoxide reductase chain B | -2.9 |
| **DNA metabolism** |  |  |  |
| APL_0817 | *xseA* | putative exodeoxyribonuclease VII large subunit | 2.82 |
| APL_1805 | *xerC* | tyrosine recombinase xerC | -11.66 |
| APL_0074 | *recR* | recombination protein RecR | -4.53 |
| APL_1170 | *priB* | primosomal replication protein | -3.83 |

| **DNA metabolism** |  |  |  |
| --- | --- | --- | --- |
| APL_0473 | *polA* | DNA polymerase I (POL I) | -3.72 |
| APL_1302 | *ligA* | DNA ligase | -2.55 |
| APL_1143 | *recA* | recombinase A | -2.52 |
| APL_0494 | *rdgC* | recombination-associated protein RdgC | 3.7 |
| APL_0459 | *recJ* | single-stranded-DNA-specific exonuclease RecJ | 4.39 |
| **Energy metabolism** |  |  |  |
| APL_1331 | *hyaA* | hydrogenase-2 small chain precursor | -14.04 |
| APL_1486 | *napB* | nitrate reductase cytochrome c-type subunit | -5.71 |
| APL_0857 | *sdaA* | L-serine dehydratase | -3.64 |
| APL_1091 | *aspA* | aspartate ammonia-lyase | -2.54 |
| APL_0102 | *nrfC* | nitrite reductase | -10.25 |
| APL_0101 | *nrfB* | cytochrome c-type protein NrfB precursor | -4.91 |
| APL_1333 | *hybB* | putative Ni/Fe-hydrogenase 2 b-type cytochrome subunit | -4.14 |
| APL_1035 | *pflA* | pyruvate formate-lyase 1-activating enzyme | -3.33 |
| APL_1645 | *atpC* | ATP synthase epsilon chain | -2.67 |
| APL_1652 | *atpB* | ATP synthase A chain | -2.62 |
| APL_1428 | *napG* | ferredoxin-type protein napG-like protein | -12.55 |
| APL_1431 | *napF* | ferredoxin-type protein NapF | -10.02 |
| APL_1430 | *napD* | putative napD protein | -5.28 |
| APL_1427 | *napH* | ferredoxin-type protein NapH-like protein | -4.2 |
| APL_0896 | *fdhE* | formate dehydrogenase accessory protein | -2.93 |
| APL_0687 | *dld* | D-lactate dehydrogenase | 2.86 |

| **Energy metabolism** |  |  |  |
| --- | --- | --- | --- |
| APL_2011 | *aldA* | Putative aldehyde dehydrogenase aldA | 6.32 |
| APL_0344 | *fruK* | 1-phosphofructokinase | -3.09 |
| APL_0230 | *gpmB* | phosphoglycerate mutase/fructose-2, 6-bisphosphatase | 5.72 |
| APL_0339 | *pepC* | phosphoenolpyruvate carboxylase | 12.05 |
| APL_0771 | *lpdA* | dihydrolipoyl dehydrogenase | 8.21 |
| APL_1737 | *sfsA* | sugar fermentation stimulation-like protein | -2.22 |
| APL_1629 | *mtlD* | mannitol-1-phosphate 5-dehydrogenase | 2.67 |
| APL_0451 | *sucD* | succinyl-CoA ligase [ADP-forming] subunit alpha | 2.85 |
| APL_1111 | *nagZ* | beta-hexosaminidase | -2.54 |
| **Lipid metabolism** |  |  |  |
| APL_1486 | *accA* | acetyl-coenzyme A carboxylase carboxyl transferase subunit alpha | -8.27 |
| APL_1993 | *fabD* | malonyl CoA-acyl carrier protein transacylase (MCT) | -2.86 |
| APL_1191 | *namA* | NADPH dehydrogenase | 2.41 |
| **Extrachromosomal element function** |  |  |  |
| APL_1058 | APL_1058 | transposase | 4.2 |
| APL_1206 | APL_1206 | plasmid stability-like protein | 8.18 |
| **Protein fate** |  |  |  |
| APL_0412 | *ecfE* | putative zinc metalloprotease | -2.05 |
| APL_1039 | *htpX* | putative protease HtpX-like protein | 2.09 |
| APL_1034 | *prlC* | oligopeptidase A | 3.03 |
| APL_0433 | *msrB* | methionine sulfoxide reductase B | -3.27 |

| **Protein synthesis** |  |  |  |
| --- | --- | --- | --- |
| APL_1785 | *rplQ* | 50S ribosomal protein L17 | -12.97 |
| APL_1769 | *rpsQ* | 30S ribosomal protein S17 | -3.89 |
| APL_1169 | *rplI* | 50S ribosomal protein L9 | -3.48 |
| APL_1972 | *rpmG* | 50S ribosomal protein L33 | -3.16 |
| APL_1821 | *rpmE* | 50S ribosomal protein L31 | 4.04 |
| APL_0654 | *alaS* | alanyl-tRNA synthetase | -3.59 |
| APL_1926 | *truD* | tRNA pseudouridine synthase D | -11.25 |
| APL_1383 | APL_1383 | tRNA (guanine-N(7)-)- methyltransferase | -4.2 |
| APL_0765 | *queA* | S-adenosylmethionine: tRNAribosyltransferase-isomerase | 2.72 |
| **Nucleotide metabolism** |  |  |  |
| APL_2018 | *purC* | Phosphoribosylaminoimidazole-succinocarboxamidesynthase (SAICAR synthetase) | 2.76 |
| APL_1172 | *purD* | phosphoribosylamine--glycine ligase | 6.21 |
| APL_1106 | *purT* | putative phosphoribosylglycinamide formyltransferase 2 | 8.47 |
| **Regulatory proteins** |  |  |  |
| APL_0048 | *arcA* | aerobic respiration control protein ArcA | -3.91 |
| APL_0133 | *cysB* | HTH-type transcriptional regulator CysB | 3 |

| **Regulatory proteins** |  |  |  |
| --- | --- | --- | --- |
| APL_1838 | *asnC* | Regulatory protein AsnC | 5.22 |
| APL_0131 | *gcvA* | glycine cleavage system transcriptional activator-like protein | 2.98 |
| APL_0395 | *rseA* | putative sigma-E factor negative regulatory protein | 2.19 |
| APL_0108 | *iclR* | putative HTH-type transcriptional regulator | -2.39 |
| APL_0059 | *narP* | nitrate/nitrite response regulator protein | 3.35 |
| APL_0571 | *gntR* | HTH-type transcriptional regulator | 14.82 |
| APL_0188 | *sixA* | possible phosphohistidine phosphatase | 5.22 |
| **Transcription** |  |  |  |
| APL_0575 | *deaD* | cold-shock DEAD box protein A-like protein | -3.37 |
| APL_0543 | *rnc* | ribonuclease III | -5.97 |
| APL_0055 | *rph* | ribonuclease PH | -3.56 |
| APL_0638 | *nusA* | transcription elongation protein NusA | -10.95 |
| APL_0201 | *nusB* | transcription antitermination protein NusB | -5.82 |
| **Transport** |  |  |  |
| APL_1991 | *brnQ* | Branched-chain amino acid transport system carrier protein braB | -7.53 |
| APL_0856 | *sdaC* | serine transporter | -6.92 |
| APL_1319 | *ptsB* | PTS system sucrose-specific EIIBC component | -5.25 |
| APL_1669 | *rbsD* | high affinity ribose transport protein RbsD | -4.43 |
| APL_1603 | *mtlA* | PTS system mannitol-specific EIICBA component | 12.84 |
| APL_0077 | *exbD2* | biopolymer transport protein ExbD2 | -7.52 |

| **Transpor**t |  |  |  |
| --- | --- | --- | --- |
| APL_0564 | *afuB_2* | Ferric transport system permease protein | -5.82 |
| APL_0276 | *frpB* | iron-regulated outer membrane protein B | -4.78 |
| APL_0128 | *yfeC* | putative iron transport system membrane protein | -2.21 |
| APL_0160 | *cbiO* | predicted ABC transport ATP-binding protein CbiO | 6.33 |
| APL_0374 | *glpF* | glycerol uptake facilitator protein | 6.77 |
| APL_0335 | *ptsN* | PTS system, nitrogen regulatory IIA-like protein | -2.56 |
| APL_0078 | *exbB2* | biopolymer transport protein ExbB2 | -4.83 |
| APL_0068 | *dppF* | dipeptide transport ATP-binding protein DppF | 3.33 |
| **Unclassified and unknowns** |  |  |  |
| APL_1636 | APL_1636 | hypothetical protein | -7.21 |
| APL_1382 | APL_1382 | hypothetical protein | -4.74 |
| APL_0519 | APL_0519 | predicted phage minor tail protein | -4.61 |
| APL_0104 | APL_0104 | autotransporter adhesin | -4.46 |
| APL_0438 | APL_0438 | hypothetical protein | -3.98 |
| APL_0196 | APL_0196 | hypothetical protein | -3.88 |
| APL_0905 | APL_0905 | hypothetical protein | -3.67 |
| APL_1891 | APL_1891 | hypothetical protein | -3.63 |
| APL_2043 | APL_2043 | hypothetical protein | -3.45 |

| **Unclassified and unknowns** |  |  |  |
| --- | --- | --- | --- |
| APL_1285 | APL_1285 | hypothetical protein | -3.24 |
| APL_1231 | APL_1231 | hypothetical protein | -3.19 |
| APL_0949 | APL_0949 | hypothetical protein | -3.12 |
| APL_0602 | APL_0602 | hypothetical protein | -3.09 |
| APL_1983 | APL_1983 | hypothetical protein | -3.06 |
| APL_0305 | APL_0305 | hypothetical protein | -3.01 |
| APL_0232 | APL_0232 | hypothetical protein | -2.74 |
| APL_0264 | APL_0264 | putative ABC transporter ATP-binding protein | -2.67 |
| APL_1272 | APL_1272 | predicted transcriptional accessory protein | -2.53 |
| APL_0363 | APL_0363 | hypothetical protein | -2.24 |
| APL_0355 | *proP* | bicyclomycin resistance-like protein | -2.18 |
| APL_0676 | APL_0676 | hypothetical protein | -2.08 |
| APL_0668 | APL_0668 | hypothetical protein | -11.77 |
| APL_1807 | APL_1807 | hypothetical protein | -8.69 |
| APL_1867 | APL_1867 | hypothetical protein | -7.97 |
| APL_1632 | APL_1632 | predicted transcriptional regulator of sugar metabolism | -7.89 |
| APL_0293 | APL_0293 | putative type I site-specific restriction-modification system, R (restriction) subunit | -7.36 |
| APL_0707 | APL_0707 | hypothetical protein | -7.1 |
| APL_1660 | APL_1660 | mannose permease IID component | -7.01 |
| APL_0937 | APL_0937 | hypothetical protein | -6.36 |

| **Unclassified and unknowns** |  |  |  |
| --- | --- | --- | --- |
| APL_0843 | APL_0843 | hypothetical tRNA/rRNA methyltransferase | -6.25 |
| APL_1573 | APL_1573 | 2,3-diketo-L-gulonate reductase | -6.03 |
| APL_1115 | APL_1115 | hypothetical protein | -5.85 |
| APL_0637 | APL_0637 | hypothetical protein | -5.4 |
| APL_0695 | APL_0695 | hypothetical protein | -5.4 |
| APL_0029 | APL_0029 | ABC transporter periplasmic protein | -5.07 |
| APL_0217 | APL_0217 | hypothetical protein | -4.11 |
| APL_0738 | APL_0738 | hypothetical protein | -3.44 |
| APL_1740 | APL_1740 | Probable Fe(2+)-trafficking protein | 2.11 |
| APL_1266 | APL_1266 | putative dehydrogenase | 2.24 |
| APL_0313 | APL_0313 | deoxyguanosinetriphosphate triphosphohydrolase-like protein | 2.24 |
| APL_1746 | APL_1746 | hypothetical protein | 2.28 |
| APL_0703 | APL_0703 | hypothetical ATP-dependent helicase | 2.3 |
| APL_0885 | APL_0885 | hypothetical protein | 2.44 |
| APL_0991 | APL_0991 | hypothetical protein | 2.57 |
| APL_1980 | APL_1980 | hypothetical protein | 2.65 |
| APL_0781 | APL_0781 | putative ATP-dependent helicase | 2.69 |
| APL_0858 | APL_0858 | hypothetical protein | 2.7 |

| **Unclassified and unknowns** |  |  |  |
| --- | --- | --- | --- |
| APL_1976 | *yedF* | hypothetical protein | 2.77 |
| APL_0672 | APL_0672 | hypothetical protein | 2.85 |
| APL_1903 | APL_1903 | hypothetical protein | 2.98 |
| APL_0788 | APL_0788 | transcriptional regulatory protein | 3.02 |
| APL_1656 | APL_1656 | hypothetical protein | 3.03 |
| APL_1044 | APL_1044 | hypothetical protein | 3.1 |
| APL_1491 | APL_1491 | hypothetical protein | 3.11 |
| APL_1855 | APL_1855 | hypothetical protein | 3.19 |
| APL_1088 | APL_1088 | hypothetical protein | 3.21 |
| APL_0162 | APL_0162 | putative phosphatase | 3.27 |
| APL_0811 | APL_0811 | hypothetical protein | 3.35 |
| APL_1141 | APL_1141 | hypothetical protein | 3.36 |
| APL_1187 | APL_1187 | hypothetical protein | 3.37 |
| APL_1167 | APL_1167 | hypothetical protein | 3.41 |
| APL_0511 | APL_0511 | hypothetical protein | 3.62 |
| APL_0787 | APL_0787 | putative ATPase | 3.71 |
| APL_1247 | APL_1247 | hypothetical protein | 3.73 |
| APL_1996 | *rssA* | hypothetical protein | 3.78 |
| APL_0480 | APL_0480 | hypothetical protein | 3.83 |

| **Unclassified and unknowns** |  |  |  |
| --- | --- | --- | --- |
| APL_1267 | APL_1267 | hypothetical protein | 4.09 |
| APL_0426 | APL_0426 | hypothetical protein | 4.15 |
| APL_1948 | APL_1948 | hypothetical protein | 4.28 |
| APL_0500 | APL_0500 | hypothetical protein | 4.41 |
| APL_1415 | APL_1415 | hypothetical protein | 4.48 |
| APL_0781 | APL_0781 | putative ATP-dependent helicase | 4.83 |
| APL_1262 | APL_1262 | transcriptional regulator MerR family | 5.15 |
| APL_1234 | APL_1234 | hypothetical protein | 5.23 |
| APL_1681 | APL_1681 | hypothetical protein | 5.74 |
| APL_1495 | APL_1495 | putative transcriptional regulator | 5.75 |
| APL_0526 | APL_0526 | hypothetical protein | 6.16 |
| APL_1588 | APL_1588 | predicted TRAP transporter solute receptor | 6.4 |
| APL_1252 | APL_1252 | hypothetical protein | 7.34 |
| APL_0882 | APL_0882 | hypothetical protein | 7.37 |
| APL_0137 | APL_0137 | hypothetical protein | 8.01 |
| APL_0222 | APL_0222 | putative lipoprotein | 10.01 |
| APL_0940 | APL_0940 | hypothetical protein | 10.79 |
| APL_0703 | APL_0703 | hypothetical ATP-dependent helicase | 12.19 |
| APL_1263 | APL_1263 | hypothetical protein | 18.51 |
| APL_1015 | *deoC* | deoxyribose-phosphate aldolase | -3.03 |
| APL_1037 | *focA* | putative formate transporter | -2.24 |
| APL_1654 | *gidB* | methyl transferase GidB | -4.41 |
| APL_0132 | *APL_0132* | putative haloacid dehalogenase-like hydrolase | 5.2 |
| APL_0403 | *engA* | GTP-binding protein EngA | 3 |
| APL_1655 | *gidA* | tRNA uridine 5-carboxymethylaminomethyl modification enzyme GidA | -2.27 |
| APL_1977 | *yedE* | hypothetical protein | 3.64 |
